# Differentiation and functioning of the lateral line organ in zebrafish require Smpx activity

**DOI:** 10.1038/s41598-024-58138-z

**Published:** 2024-04-03

**Authors:** Alberto Diana, Anna Ghilardi, Luca Del Giacco

**Affiliations:** Department of Biosciences, Università degli Studi di Milano, Via Celoria 26, 20133 Milan, Italy

**Keywords:** Ciliogenesis, Developmental biology, Disease model

## Abstract

The small muscle protein, X-linked (*SMPX*) gene encodes a cytoskeleton-associated protein, highly expressed in the inner ear hair cells (HCs), possibly regulating auditory function*.* In the last decade, several mutations in *SMPX* have been associated with X-chromosomal progressive non syndromic hearing loss in humans and, in line with this, Smpx-deficient animal models, namely zebrafish and mouse, showed significant impairment of inner ear HCs development, maintenance, and functioning. In this work, we uncovered *smpx* expression in the neuromast mechanosensory HCs of both Anterior and Posterior Lateral Line (ALL and PLL, respectively) of zebrafish larvae and focused our attention on the PLL. Smpx was subcellularly localized throughout the cytoplasm of the HCs, as well as in their primary cilium. Loss-of-function experiments, via both morpholino-mediated gene knockdown and CRISPR/Cas9 F0 gene knockout, revealed that the lack of Smpx led to fewer properly differentiated and functional neuromasts, as well as to a smaller PLL primordium (PLLp), the latter also Smpx-positive. In addition, the kinocilia of Smpx-deficient neuromast HCs appeared structurally and numerically altered. Such phenotypes were associated with a significant reduction in the mechanotransduction activity of the neuromast HCs, in line with their positivity for Smpx. In summary, this work highlights the importance of Smpx in lateral line development and, specifically, in proper HCs differentiation and/or maintenance, and in the mechanotransduction process carried out by the neuromast HCs. Because lateral line HCs are both functionally and structurally analogous to the cochlear HCs, the neuromasts might represent an invaluable—and easily accessible—tool to dissect the role of Smpx in HCs development/functioning and shed light on the underlying mechanisms involved in hearing loss.

## Introduction

Human *SMPX* gene was first identified in 1999 when its whole genomic structure was revealed to be made of five exons and four introns, spanning a region of about 52.1 kb^1^. The gene encodes a 9 kDA prolin-rich protein of 88 aa that contains two casein kinase II phosphorylation sites (CKII), a PEST sequence, and a nuclear localization signal (NLS)^2–4^. Human *SMPX* was initially reported to be predominantly expressed in both skeletal and cardiac muscle, whilst the murine *Smpx* exhibited a more complex pattern of expression as in that its expression was described also in the liver, testis, kidney and brain^1^.

In the last decade, mutations in the *SMPX* gene have been associated with nonsyndromic X-linked hearing loss DFNX4^4–14^. Interestingly, *SMPX* was shown to be abundantly expressed in both fetal and adult human inner ear^4^, while mouse *Smpx* was concordantly expressed with the master regulator of inner ear hair cell (HC) specification, the transcription factor *Atoh1,* in all vestibular organs and all cochlear turns of the developing mouse inner ear, thus suggesting a Smpx putative role during HC differentiation and/or functioning^15^. Furthermore, HC development was found to be severely affected in zebrafish embryos lacking Rbm24a, an RNA-binding protein that directly regulates *smpx* mRNA stability^16^. As we previously demonstrated, the zebrafish Smpx protein inside the inner ear HC was mainly localized at the level of the cuticular plate (CP), an actin-rich structure that is necessary for the correct development of both stereocilia and kinocilia, and to carry out mechanotransduction^17^. Employing a zebrafish model of Smpx deficiency, we showed that the protein exerted a primary function in the proper structural organization of the inner ear HCs sensory bundle as in that both stereocilia and kinocilia displayed evident morphological alterations in terms of number and length^11^, as later also confirmed in a knock-out mouse model^18^. Moreover, we demonstrated that inner ear HCs of Smpx-deficient embryos lack of mechanotransduction activity, providing the first possible explanation for the reported cases of *SMPX*-linked hearing loss in human patients^4–14^. In this work we focused our attention on the zebrafish lateral line, a sensory system positioned on the outside of the organism, which grants the animal the ability to detect water flow changes in terms of motion and pressure^19^. The lateral line organ forms, together with the inner ear, the octavolateralis sensory system of fishes^20^. The functional units of the lateral line are the neuromasts, a series of sensory organs arrayed along the head and body of the fish. Each neuromast contains a central cluster of HCs which are structurally and functionally analogous to the HCs of the cochlea, but more easily accessible than the latter^19,21,22^. During development, the first neuromasts are formed through the rostrocaudal migration of the posterior lateral line primordium (from now on PLLp)—namely a transient structure generated from placodal cells adjacent to the ear—occurring between 24 and 40 h post fertilization (hpf)^19,23^. At around 42 hpf, other posterior lateral line primordia (primII and primD) are formed and their migration will give rise to accessory/secondary neuromasts^24^. Here, we describe the activity of zebrafish *smpx*, that is expressed in all neuromasts of the PLL. We also depicted the distribution of the encoded protein in the neuromast, which occurred exclusively in the HCs, throughout the cytoplasm and the kinocilium of the cells. Also, by means of loss-of-function experiments, we elucidated the role played by the gene in controlling the proper development and function of the neuromasts. Indeed, lack of Smpx was associated with a decrease in size of the PLLp (in which the Smpx protein was also present), fewer HCs-positive neuromasts, reduced density of kinocilia per neuromast, the alteration of the kinocilium structure and, finally, the substantial impairment in the mechanotransduction activity of the neuromast HCs.

## Results

### *smpx* expression in the neuromasts

**Figure 1:**
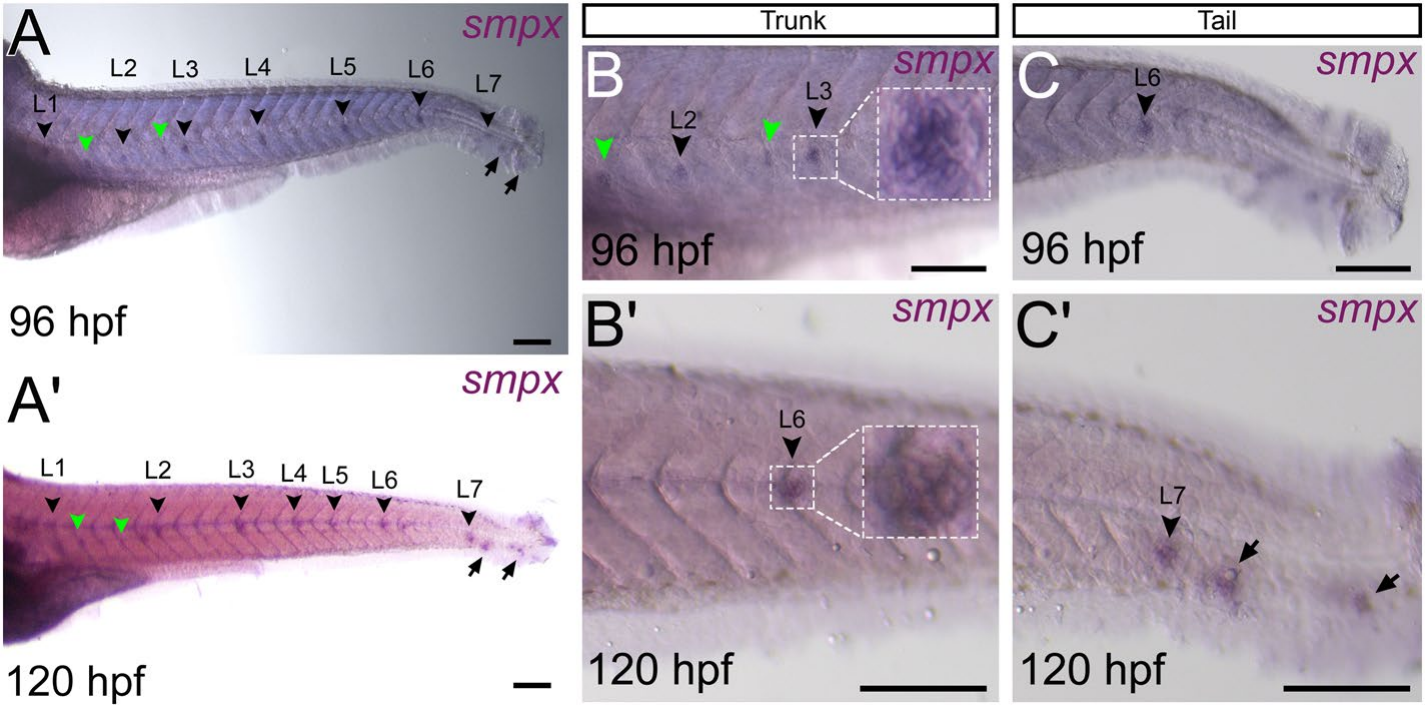
*smpx* expression in the neuromasts of the posterior lateral line. Representative stereomicroscope images of *in-situ* hybridizations performed with the *smpx* riboprobe on whole mount larvae at 96 and 120 hpf. (**A**,**A’**) The gene is expressed in all the neuromasts (L1-L7, black arrowheads) establishing the lateral line at both stages analyzed. The *smpx*-specific signal is present also in the accessory neuromasts (green arrowheads) and in the terminal neuromasts of the tail (black arrows). (**B**,**B’**) Detailed view of *smpx* signal in the L2, L3 and L6 neuromasts of the trunk at 96 and 120 hpf. The white-dotted rectangles show magnified neuromasts with the typical rosette-like structure, the green arrowheads indicate the accessory neuromasts. (**C**,**C’**) Detailed view of *smpx* signal in the L6, L7, and terminal neuromasts (black arrows) of the tail at 96 and 120 hpf. (**A**,**B**) Neuromasts appear slightly below the median line because they were flat-mounted under a coverslip. Lateral views, anterior to the left. Scale bars are 160 µm.

Through an *in-situ* hybridization protocol with slight modifications to avoid potential neuromasts degradation, the *smpx*-specific transcripts were detected in the zebrafish neuromast cells (Fig. 1). The first evidence of *smpx* expression occurred at 96 hpf, where the signal was clearly visible in the cells of the deposited neuromasts of the head (data not shown), and along the trunk and tail of the larva (Fig. 1A). At 120 hpf, the *smpx*-specific signal was observed in all neuromasts of the posterior lateral line (PLL, Fig. 1A’). At 96 hpf and 120 hpf, we counted 7 neuromasts of the PLL per each side of the larva (Fig. 1A,A’) with the typical rosette-like structure (Fig. 1B,B’), plus 1–2 accessory neuromasts and 2 terminal neuromasts at the end of the tail (Fig. 1C,C’). A barely distinguishable signal was observed also at 48 and 72 hpf (data not shown), suggesting that the gene was already expressed at these earlier developmental stages, as confirmed by the presence of the protein since 48 hpf (see below).

### Smpx localization in the neuromast hair cells

**Figure 2:**
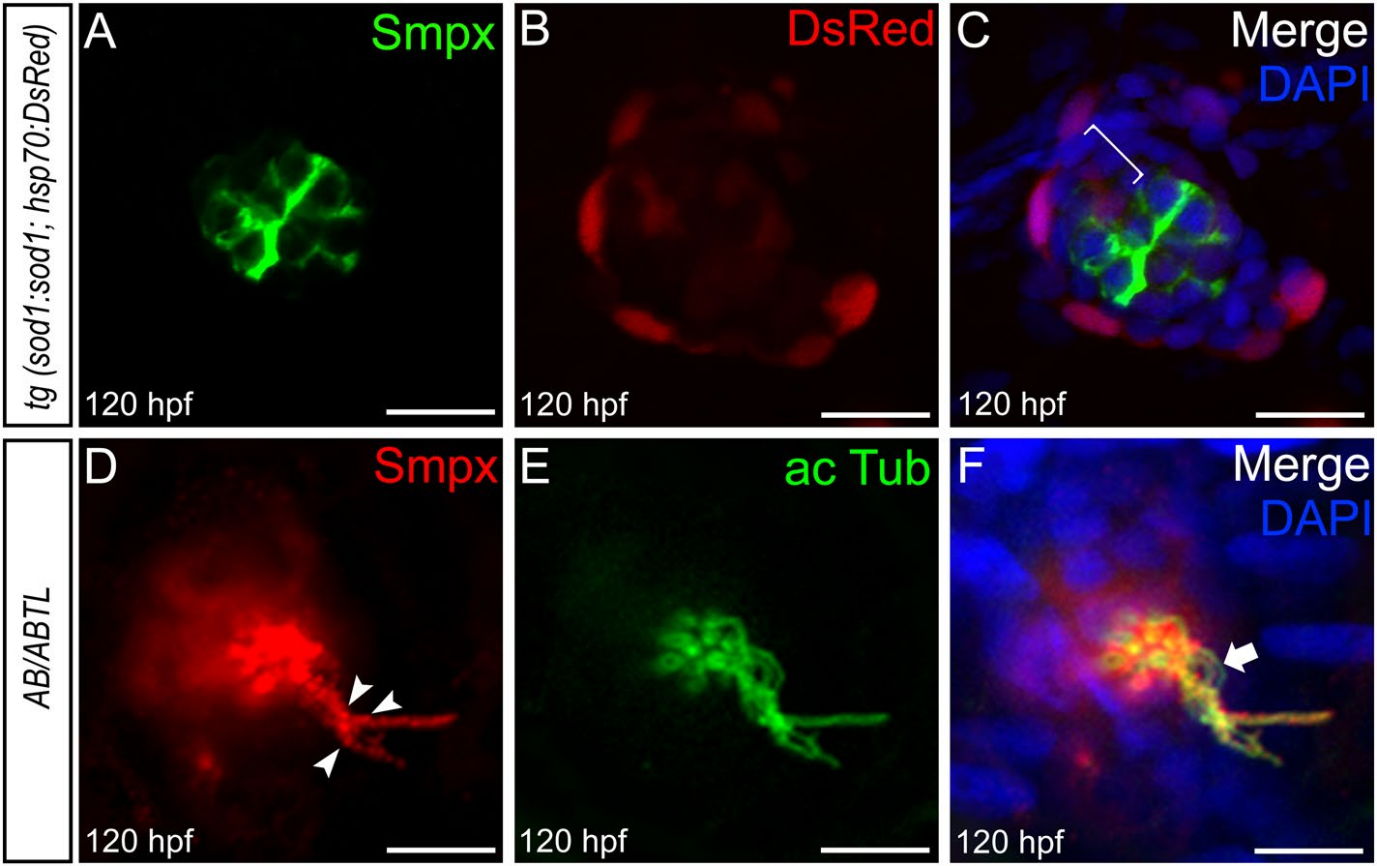
Smpx protein localization in the neuromast mechanosensory hair cells. Confocal Z-stacks of immunofluorescence on whole mount 120 hpf larvae. (**A**–**C**) Smpx (green) localizes in the most internal cells of the neuromast, namely the mechanosensory hair cells, while it is excluded from the outermost cells (red) of the transgenic line *tg (sod1:sod1; hsp70:DsRed*) and the presumptive accessory cell layers (white bracket) encompassed in between the outmost DsRed-positive cells and the inmost hair cells. (**D**–**F**) Smpx (red) co-localizes with acetylated tubulin (green) in the kinocilium, with Smpx showing a ‘beads-on-a-string’ distribution (arrowheads in D). In some cases, the kinocilium is not labeled by the anti-Smpx antibody (arrow in F). (**A**–**F**) Larvae were counterstained with DAPI (blue) to visualize cell nuclei. Lateral views, anterior to the left. Scale bars are 15 µm in **A**–**C** and 10 µm in **D**–**F**.

The *in-situ* hybridization data regarding the expression of *smpx* in the neuromasts were fully confirmed at the protein level. Indeed, whole mount immunofluorescence experiments with an anti-Smpx polyclonal antibody (Supp. Fig. S1), proved the localization of Smpx in the neuromasts of the PLL at 48 (Supp. Fig. S1A), 72 (Supp. Fig. S1B), 96 (Supp. Fig. S1C), 110 (Supp. Fig. S1D), and 120 hpf (Supp. Fig. S1E,F). Moreover, the Smpx protein was also localized at the level of the head neuromasts (Supp. Fig. S1B-E) that form the anterior lateral line (ALL), a structure that develops independently from the PLL^25^. In all 120 hpf neuromasts, Smpx localized quite exclusively in the cytoplasm of the cells (Supp. Fig. S1F, Fig. 2, Supp. Fig. S2) and never in the nucleus, in contrast to an earlier study that found the protein accumulated within the nucleus of C2C12 cells^2^. By employing the transgenic zebrafish line *tg (sod1:sod1; hsp70:DsRed)*^26^, in which the red fluorescence mainly paints the outermost cells of the neuromasts (Supp. Fig. S3), we precisely localized Smpx in the innermost cluster of cells (Fig. 2A) and not in the DsRed-positive external cell layer (Fig. 2B), as demonstrated by the lack of overlapping signals (Fig. 2C). Furthermore, it is clear the absence of Smpx in the presumptive accessory cells’ territory, encompassed in between the DsRed-positive outer layer and the inmost cells of the neuromast (Fig. 2C). In addition to displaying a massive signal throughout the cytoplasm, Smpx was strongly present in the microtubule-based kinocilium (Fig. 2D-F, Supp. Fig. S2). The distribution of the signal appeared quite uniform in some hair cells, while in others the protein become visible in a ‘beads-on-a-string’ configuration, or not visible at all (Fig. 2F). The Smpx localization in the primary cilium strongly supports the observation that the Smpx-positive cells are the actual mechanosensory HCs responsible for the conversion of mechanical stimuli into electric signal^27^. This data is in line to what we previously reported in the inner ear, where Smpx was localized exclusively in the HCs of the sensory patches^11,17^.

### The number of intact PLL neuromasts is affected by lack of Smpx

**Figure 3:**
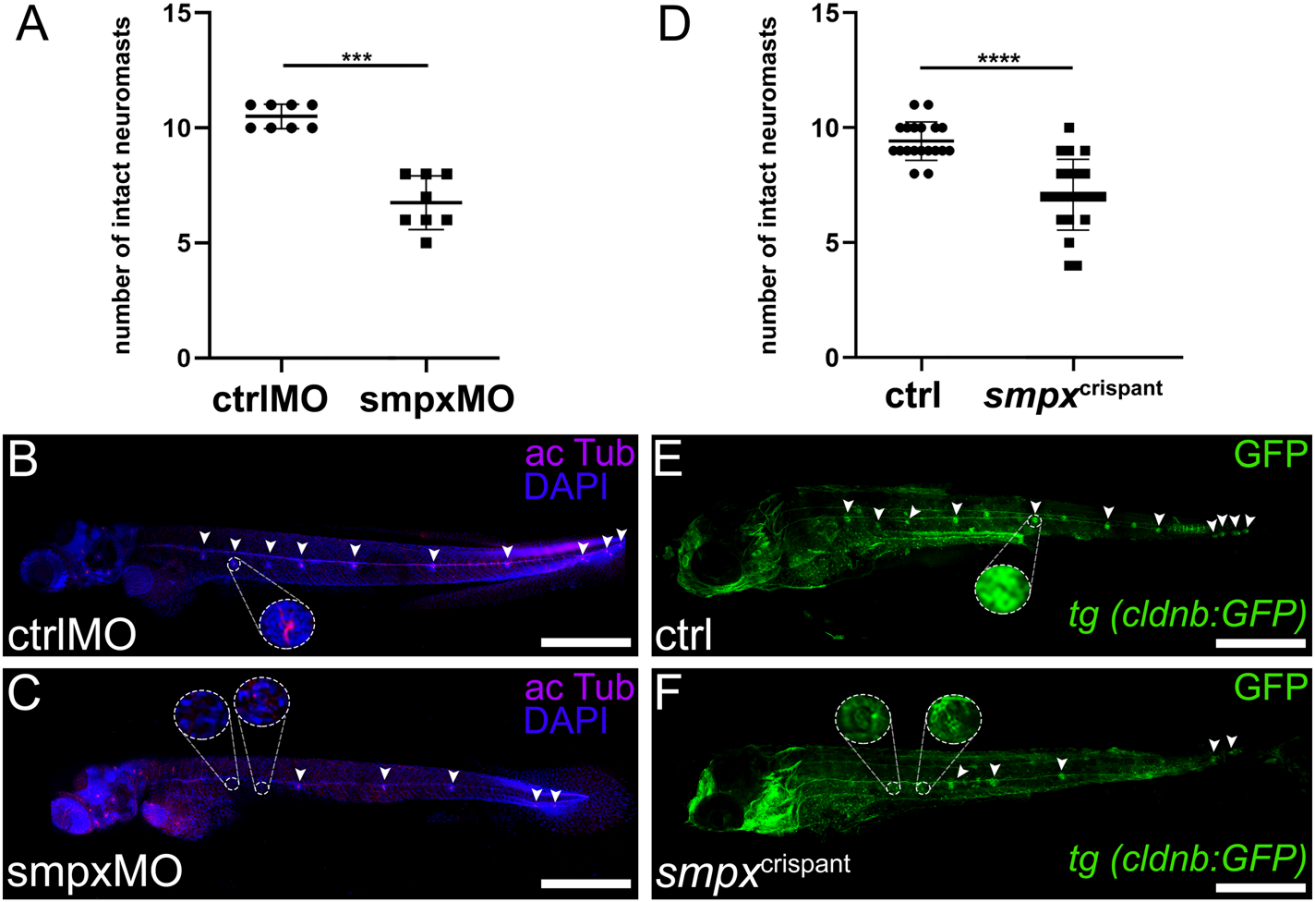
Smpx-deficiency leads to a lower number of intact neuromasts in morphant and crispant larvae. (**A**) Quantification of the reduced number of intact neuromasts in smpxMO versus ctrlMO larvae. (**B**,**C**) Representative confocal images of ctrlMO and smpxMO larvae labeled with an anti-acetylated tubulin antibody (magenta). Larvae were counterstained with DAPI to visualize cell nuclei. Insets in C show magnified neuromasts lacking the innermost nuclei and the knocilia of the HCs (compare to inset in B). (**D**) Quantification of the reduced number of intact neuromasts in *smpx*^crispant^ versus ctrl larvae. (**E**,**F**) Representative confocal images of immunofluorescence experiments with an anti-GFP antibody in ctrl and *smpx*^crispant^ *tg (cldnb:GFP)* transgenic larvae. Insets in F display magnified neuromasts showing the depletion of the innermost cell cluster (compare to inset in E). (**A**,**D**) Unpaired *t*-test was performed to assess statistical significance by setting *p* ≤ 0.05 (*), *p* ≤ 0.01 (**), *p* ≤ 0.001 (***) and *p* ≤ 0.0001 (****) as significant. (**A**) ctrlMO n = 8, smpxMO n = 8. (**D**) ctrl n = 19, *smpx*^crispant^ n = 22. (**B**,**C** and **E**,**F**) Confocal Z-stacks images taken from 120 hpf whole mount larvae; lateral views, anterior to the left. Scale bars are 500 µm.

The role of Smpx in proper neuromast development was analyzed, at first, by gene knockdown through the microinjection of a previously validated ATG-targeting *smpx*-specific morpholino^11^, then by CRISPR/Cas9-mediated F0 gene knockout. Being Smpx expressed in the mechanosensory HCs of the neuromasts, we employed the FM 4–64 vital dye to stain such cells in vivo^28,29^. The reduction in number of FM 4–64-positive HCs clusters was observed in the morphant larvae (data not shown) and confirmed in the *smpx* F0 knockout model (*smpx*^crispants^, Supp. Fig. S4A-C). To gain insight into these observations, we performed a DAPI/acetylated tubulin co-staining in *smpx* morphant and control embryos, revealing a diminished number of acetylated tubulin-positive neuromasts in the entire PLL (Fig. 3A-C), and the presence of hole-like structures surrounded by nuclei in the center of the regions where homeostatic neuromasts should be deposited (insets in Fig. 3B,C). To further increase our confidence, we carried out an anti-GFP staining in the *smpx*^crispant^ larvae generated in the *tg (cldnb:GFP)* transgenic line background, where the GFP is expressed in all neuromast cell types^30^; the IHC on such transgenic line was performed to amplify the low fluorescence signal detected in live larvae, that represented a major setback for the analyses*.* The number of intact neuromasts appeared lower also in the *smpx*^crispants^, supporting our above-mentioned observations (Fig. 3D-F). As for the previous case, the hypothesized lack of HCs from the center of the neuromasts left hole-like structures encircled by the other cell types (insets in Fig. 3E,F). Finally, *smpx* deletion performed on the *tg (sod1:sod1; hsp70:DsRed)* line revealed that the outer cell layer, and in turn, the overall number of PLLp-derived neuromasts, was unaltered by the lack of the protein (Supp. Figure 4D-F), confirming that Smpx deficiency does not affect all the neuromast cell types.

### Smpx is expressed in the posterior lateral line primordium and controls its size

**Figure 4:**
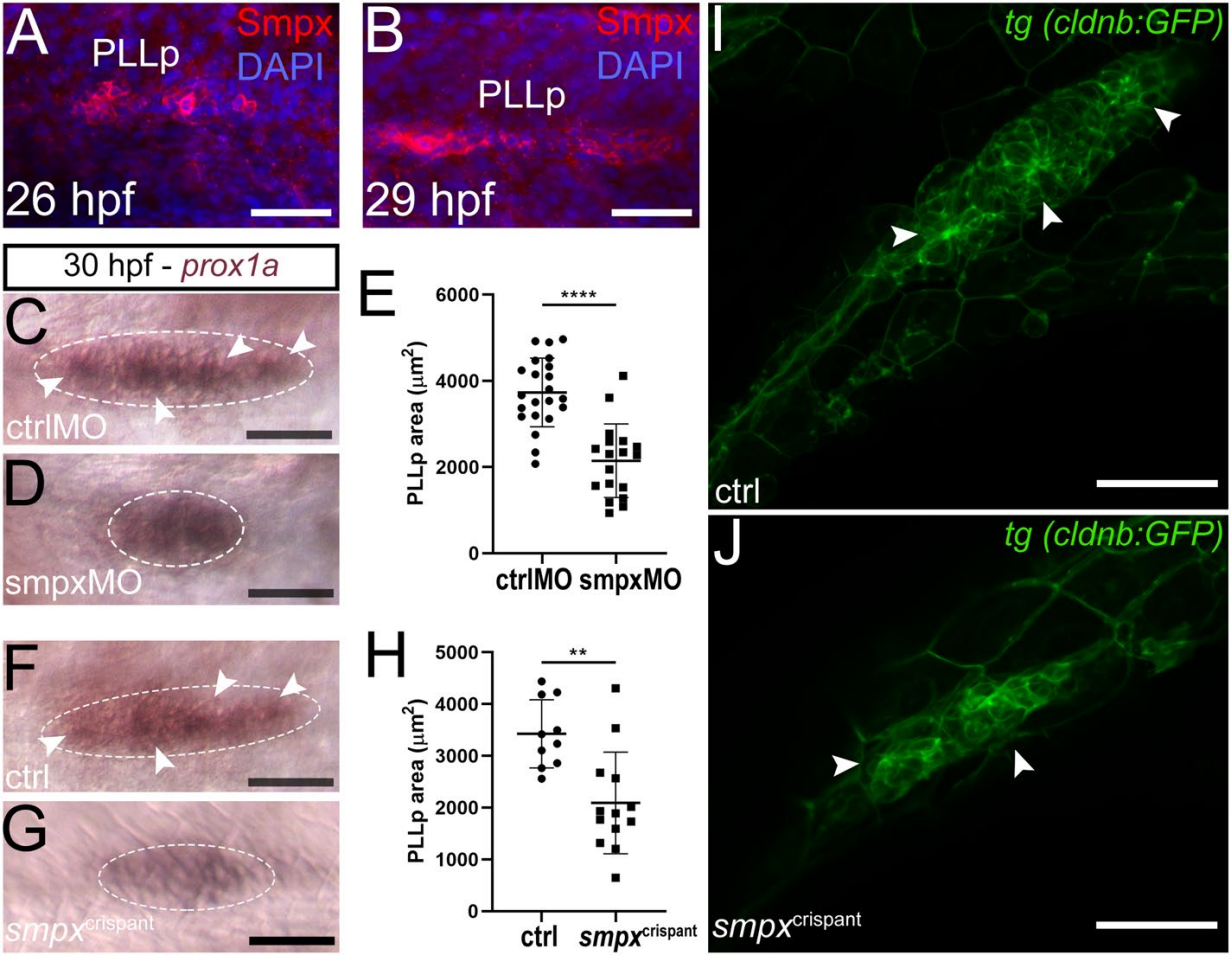
Smpx-deficiency causes the decrease in the PLLp size. (**A**,**B**) Immunofluorescence with the anti-Smpx antibody unveiling the localization of the protein in the PLLp at 26 and 29 hpf. (**C**,**D**) *In*-*situ* hybridization with a *prox1a* probe to compare the PLLp size (white-dotted encircled areas) in 30 hpf ctrlMO and smpxMO; the PLLp of Smpx-deficient embryos appears smaller in size; the typical rosette-like structures present in the ctrlMO sample (white arrowheads in C) are not clearly distinguishable in the smpxMO sample. (**E**) Size quantification in ctrlMO and smpxMO showing a drastic decrease in the PLLp area following the downregulation of *smpx.* (**F**,**G**) *In*-*situ* hybridization with a *prox1a* probe to compare the PLLp size (white-dotted encircled areas) in 30 hpf ctrl and *smpx*^crispant^ embryos; the PLLp of the *smpx*^crispant^ embryos appears smaller in size and the typical rosette-like structures present in the ctrl sample (white arrowheads in F) are not clearly distinguishable in the *smpx*^crispant^ preparation. (**H**) Size quantification in ctrlMO and *smpx*^crispant^ showing a drastic decrease in the PLLp area following the ablation of *smpx.* (**I**,**J**) Z-stack confocal images of the PPLp at 30 hpf in *tg (cldnb:GFP)* embryos; the shape of the epithelial rosettes in control embryos are lost in *smpx*^crispants^. (**A**,**B**) Confocal Z-stacks taken from whole mount embryos; lateral views, anterior to the left. Embryos were counterstained with DAPI to visualize cell nuclei. (**E**,**H**) Unpaired *t*-test was performed to assess statistical significance by setting *p* ≤ 0.05 (*), *p* ≤ 0.01 (**), *p* ≤ 0.001 (***) and *p* ≤ 0.0001 (****) as significant. (**E**) ctrlMO n = 22, smpxMO n = 18. (**H**) ctrl n = 10, *smpx*^crispant^ n = 13. Scale bars are 50 µm.

Because PLL proper development was severely altered by Smpx-deficiency, we decided to study earlier steps of the organ assembly analyzing the migrating PLL primordium (PLLp). Interestingly, we found that Smpx localized also in this structure, starting from ~ 26 hpf (Fig. 4A,B). To test whether the lack of Smpx might cause an improper shaping of the PLLp, eventually leading to a defective deposition of neuromast cell precursors as development proceeds, we investigated the overall morphology of the PLLp following *smpx* downregulation/knockout. The *prox1a* RNA probe, a well-known marker for neuromast hair and supporting cells^31^, was used to visualize the PLLp, which appeared smaller in size as a consequence of Smpx deficiency (Fig. 4C,D). Indeed**,** the quantification of the *prox1a*-positive PLLp area showed that *smpx* downregulation caused a significant reduction of 1585 ± 261.8 µm^2^ (Fig. 4E), strongly supporting the above-mentioned hypothesis. The same outcome was obtained through *smpx* knockout (Fig. 4F,G), with a significant reduction of the PLLp area of 1337 ± 361.2 µm^2^ (Fig. 4H). Moreover, because *prox1a* is a selective HC/supporting cell marker, its expression permits the visualization of the rosette-like structures which guarantee the proper formation and deposition of protoneuromasts during PLL organ morphogenesis. Interestingly, such structures were not detectable in *smpx* morphants (compare Fig. 4C,D) and crispants (compare Fig. 4F,G). Through an in-depth analysis of the *tg (cldnb:GFP)* line, the rosette-like structures of the PLLp in *smpx*^crispants^ appeared numerically less dense and structurally altered compared to the normal organization of the controls (Fig. 4I,J).

### The mechanotransduction activity of the neuromast ciliated cells depends on Smpx

**Figure 5:**
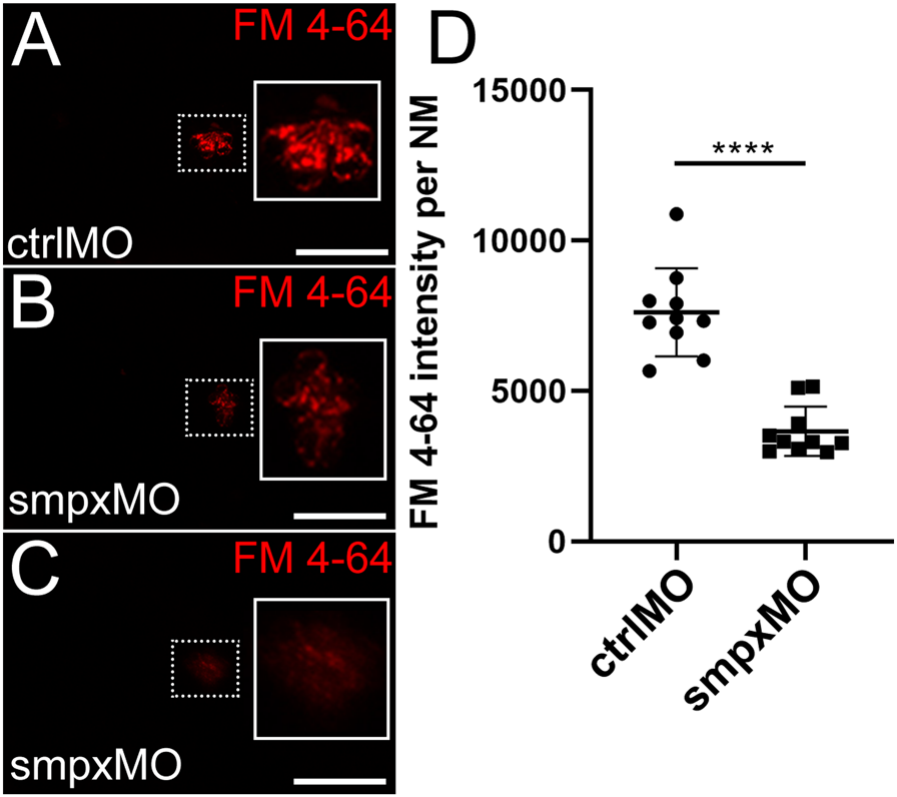
Smpx-deficiency causes a reduced mechanotransduction activity of the neuromast hair cells. (**A**–**C**) Representative images of FM 4–64 incorporation in one control neuromast (ctrlMO) and two Smpx-deficient (smpxMO) neuromasts at 120 hpf (white rectangles on the right of each single image are magnifications of the corresponding, white-dotted rectangles on the left); compared to the control (**A**), *smpx*-morphants (**B**,**C**) display a reduced FM 4–64 dye uptake. (**D**) Quantification of the FM 4–64 dye uptake calculated as fluorescence intensity per each neuromast (n = 10 for both controls and morphants). (**A**–**C**) Confocal Z-stacks taken from whole mount larvae. (**D**) Unpaired *t*-test was performed to assess statistical significance by setting *p* ≤ 0.05 (*), *p* ≤ 0.01 (**), *p* ≤ 0.001 (***) and *p* ≤ 0.0001 (****) as significant. Scale bars are 50 µm.

Kindt and colleagues demonstrated that neuromast HCs require kinocilia for proper mechanosensation^32^. In line with the evidence that Smpx localizes also along the kinocilium, we tested the role of the protein in lateral line mechanotransduction by means of gene loss-of-function. Smpx downregulation caused a reduction in the mechanotransduction activity of the deposited neuromast HCs, at 120 hpf, as demonstrated by incubating live larvae in embryo water containing the FM 4–64 vital dye. Indeed, the lower mechanotransduction activity was highlighted by the defective uptake of the dye (Fig. 5A–C), resulting in a significant decrease in the overall fluorescence intensity in *smpx* morphant larvae compared to controls (Fig. 5D).

### Smpx is required for normal kinocilium structure

**Figure 6:**
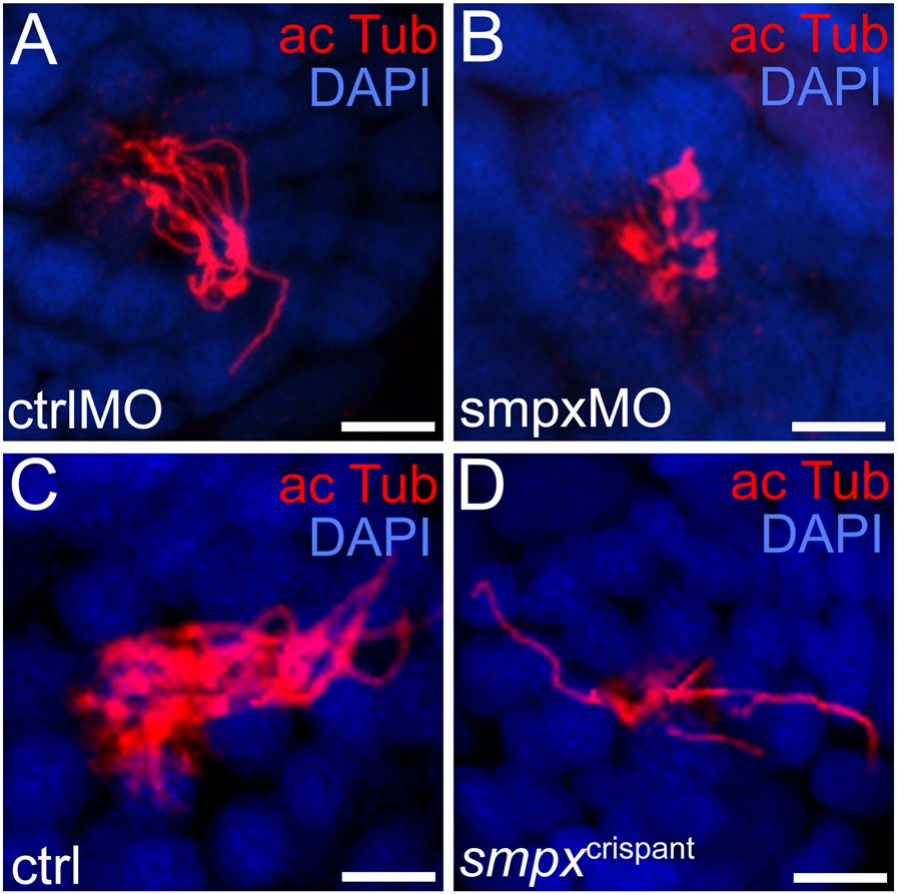
Smpx is required for normal kinocilium structure. Immunofluorescence performed on whole mount 120 hpf larvae with an antibody against acetylated tubulin to label the neuromast kinocilia. (**A**,**B**) Compared to controls (ctrlMO), the kinocilia of morphant larvae (smpxMO) appear clearly shorter in length and numerically less dense. (**C**,**D**) As for the knockdown experiment, when compared to controls (ctrl), the kinocilia of crispant larvae (*smpx*^crispant^) are shorter and numerically lower. (**A**,**B**) ctrlMO n = 5, smpxMO n = 4. (**C**,**D**) ctrlMO n = 5, smpxMO n = 5. (**A**–**D**) Confocal Z-stack images of neuromast L4 for each larva. n is equal to the sum of the larvae from two independent experiments. Embryos were counterstained with DAPI to visualize cell nuclei. Scale bars are 6 µm.

Based on the lack of mechanosensitivity following Smpx deficiency, we analyzed, by immunohistochemistry, both the general morphology of the primary cilium of the HC and the structure of the neuromasts. The number of kinocilia in morphant neuromasts was clearly decreased in comparison to controls, and such massive reduction was accompanied by the severe alteration in the overall length of the kinocilia (Fig. 6A,B). Again, to strengthen such evidence, we investigated the consequence of *smpx* knockout on neuromasts assembly, gaining comparable results. Indeed, in *smpx*^crispant^ larvae, the primary cilia of the neuromasts appeared dramatically less dense compared to controls (Fig. 6C,D).

## Discussion

In addition to our previous expression data^17^, whole mount *in-situ* hybridization (WISH) experiments revealed that *smpx* was also expressed in the neuromasts, namely the specialized ciliated structures located along the trunk of the animal which establish the lateral line organ^33^. The expression was present, up to the latter stage analyzed (120 hpf), in the neuromasts of the head, in those placed all along the trunk and tail of the embryo, as well as in the terminal neuromasts of the tail. In line with the mRNA expression, the protein was also localized in the neuromasts starting from 48 hpf, the earlier stage analyzed. Specifically, by employing the transgenic line *tg (sod1:sod1, hsp70:DsRed)*, that we used as a reporter line for its ability to drive DsRed expression in the outer cell layer of the neuromasts, we demonstrated that Smpx is specifically localized in the most internal cells of the ciliated structures; these cells are the actual sensors responsible for mechanosensation^34^. Strongly reinforcing our data, previous scRNA-seq experiments revealed *smpx* mRNA to be highly enriched in the transcriptome of both young and mature HCs of homeostatic neuromasts, when compared to the transcriptome profiles of supporting and mantle cells^35^. However, both our most in-depth immunofluorescence experiments for Smpx protein localization and the scRNA-seq by Lush and colleagues^35^ were performed only with 120 hpf neuromasts and it is not possible to exclude a priori the expression of *smpx* also in other neuromast cell types at different developmental stages. Quite interestingly, as clearly visible at 120 hpf, the protein was localized not only throughout the cytoplasm of the neuromast sensory HCs, but also at the level of the kinocilium. This last data is extremely surprising, because Smpx was never detectable in kinocilium of the HCs of the zebrafish larva inner ear at all stages analyzed^17^. A possible explanation for such apparent discrepancy could be represented by the different timing at which the HCs in the two districts acquire a more and more mature-type organization, as previously suggested for later stages of development^36^. Notably, some neuromasts displayed Smpx arranged in a ‘beads-on-a-string’ fashion all along the kinocilium and in each of the HC of the structure, while in other neuromasts Smpx signal painted the kinocilium of some HCs but was completely absent from others. This is very interesting considering that primary cilia are highly dynamic organelles in which their functioning is strictly dependent on ciliary proteins turnover and subcellular localization^37^. Therefore, the intense trafficking of ciliary membrane proteins from the Golgi apparatus towards ciliary compartments may represent the event underlying the heterogeneity of Smpx signal in the kinocilia observed in this work. Functional experiments via gene loss-of-function, by using both morpholino and CRISPR/Cas9 approaches, showed that the lack of Smpx led to a reduction in the number of fully formed neuromasts. Interestingly, it was recently demonstrated in homozygous zebrafish mutants for *rbm24a*—a gene encoding an RNA-binding protein that directly regulates the stability of the *smpx* transcript—a similar defective HCs morphogenesis^16^, further supporting the link between these two genes and their putative co-presence in the same pathway during neuromast HCs development/maintenance. Indeed, *smpx* was found downregulated in *rbm24a* mutants, indicating that the reason for the reduced number of neuromast HCs in the mutant larvae^16^ might be, in fact, the insufficient amount of Smpx synthesized by the HCs. On the other hand, while in *rbm24a* mutants the number of neuromasts appeared lower^16^, our experiments revealed that the overall number of deposited PLLp-derived neuromasts at 120 hpf was mostly unaffected in Smpx-deficient larvae, thus ruling out the hypothesis that *rbm24a* and *smpx* might act together also during the gross deposition of protoneuromasts. Interestingly, Smpx starts to be detectable in the PLLp of control embryos already at 26 hpf, as well as few hours later, when the PLLp starts to release the first neuromast. Thus, the localization of Smpx in the PLLp, and the defective phenotype of the lateral line organ in Smpx-deficient larvae, was suggestive that the lower number of HCs positive neuromasts could be the direct consequence of the PLLp shrinkage that takes place at earlier developmental stages. By visualizing the PLLp with the *prox1a* riboprobe and with the *tg (cldnb:GFP)* line, we observed that these structures of most Smpx-deficient embryos were largely deprived of the HCs precursors. Indeed, the classical rosette-like structures (with the precursors at their center) that form into the trailing zone of the PLLp and, in turn, assemble themselves into protoneuromasts under the control of the Fgf signaling^38,39^, were mostly absent or barely distinguishable. Although we reported a defective phenotype in both PLLp- and primII/primD-derived neuromasts, the alteration of the overall structure of the PLLp in Smpx-deficient embryos might account only for those neuromasts that are deposited from the PLLp. Therefore, further investigations will be required to assess not only whether Smpx localizes in the secondary primordia, but also to comprehend the upstream event underlying the aberrant morphology that we observed also in the accessory/secondary neuromasts. Interestingly though, the PLLp and primII/primD are formed from their respective primary and secondary placodes upon the same retinoic acid signaling during late gastrulation, suggesting a common origin and a strict interconnection^24^. Due to the prominent presence of Smpx in the neuromast primary cilia, we searched for possible structural kinocilium alteration following Smpx ablation. As expected, the HC kinocilia of Smpx-deficient neuromasts were shorter compared to those of the controls. Moreover, although it was not possible to precisely quantify the number of primary cilia because they overlapped each other, kinocilia of Smpx-deficient embryos appeared visually less dense than controls, thus suggesting a reduction in number, as occurred in the inner ear HCs^11^. The mechanism by which Smpx-deficiency causes primary cilia degeneration/developmental impairment is still unknown and remains highly speculative, thus requiring additional investigations. Nevertheless, our analysis pointed out that the lack of Smpx in the neuromast HCs caused a reduction in the mechanotransduction activity of such cells, as demonstrated by the lower FM 4–64 dye fluorescence, in line to what we observed in the inner ear HCs^11^.

**Figure 7:**
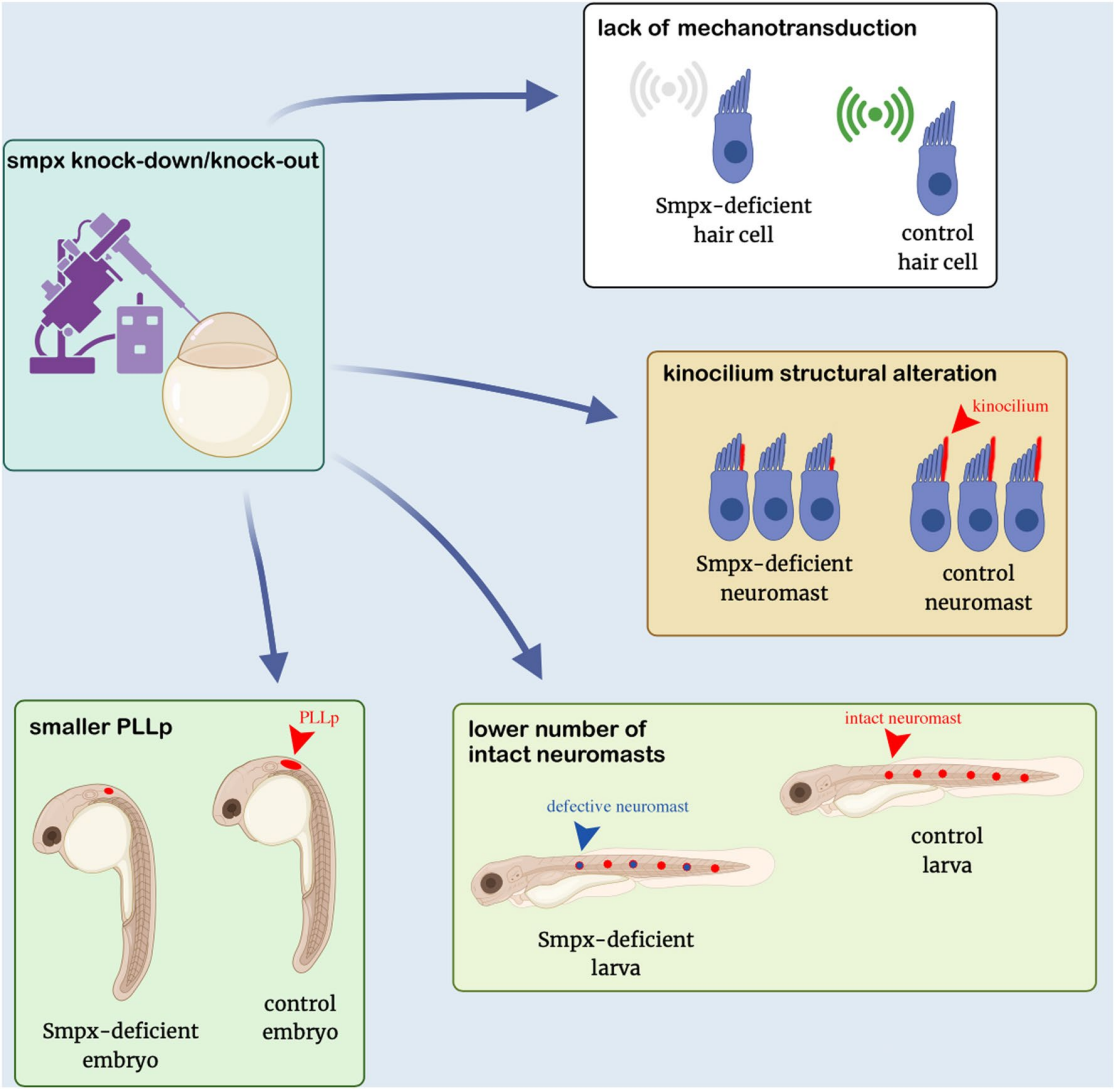
*smpx* shapes the lateral line organ and affects its mechanosensitivity. Smpx-deficiency affects the number of intact neuromasts in the PLL, possibly because of the role of the gene in directing the development of the HCs in the PLLp. Furthermore, Smpx is essential in proper kinocilium modeling and HCs mechanotransduction.

In conclusion, our study provides further evidence of the role played by *smpx* in neuromast HC development and functioning. This work highlights novel territories of *smpx* expression, namely the mechanosensory HCs of the neuromasts and the PLLp and demonstrates the central role of Smpx in controlling the shaping of the PLL sensory organ and its mechanosensitivity (Fig. 7). This will be of great interest for hearing research (zebrafish lateral line represents a useful tool to study human hearing loss^40^) as in that the neuromasts are much more accessible in terms of imaging assays and drugs administration. Because of the lack of definitive therapeutic options for genetic deafness, the combination of the knockout models under generation, the expression of *smpx* in the mechanosensory ciliated cells of the neuromasts and the functional, genetical and evolutionarily comparability of such cells with those of the human inner ear^28^ will eventually lead to the establishment of an *in-vivo* platform for fast and unbiased pharmacological screenings of novel (or repositioned) molecules capable of reverting the pathological condition.

## Materials and methods

### Zebrafish husbandry and maintenance

Fish were maintained in the facility of the University of Milan, Via Celoria 26, 20,133 Milan, at 28 °C on a 14-h light/10-h dark cycle. The temperature of the water in the aquarium was maintained between 28 °C and 28.5 °C, with the pH spanning between 6.8 and 7.5. Fish in the aquarium were fed three times per day with granular food of different dimension according to growth phases. Our facility strictly complies with the relevant European (EU Directive 2010/63/EU for animal experiments) and Italian (Legislative Decree No. 26/2014) laws, rules, and regulations (Auth. Min. 24/2019-UT), as also confirmed by the authorization issued by the municipality of Milan (PG 198,283/2019). All procedures were carried out in accordance with the relevant Italian and European guidelines and regulations, and the ARRIVE guidelines (https://arriveguidelines.org/).

### Zebrafish lines used in this work and egg collection

In the experiments involving embryos, the zebrafish lines used were as follows: wild-type of the AB and ABTL strains, *tg (sod1:sod1; hsp70:DsRed)* transgenic line^26^, and *tg (cldnb:GFP)*^30^. Embryos were collected by means of natural spawning, picked up with a plastic Pasteur pipette and staged in embryo water-containing Petri dishes for downstream analysis, according to Kimmel and co-workers^41^. To avoid pigmentation, embryos used in whole mount *in-situ* hybridization (WISH) and immunofluorescence experiments were raised in embryo water supplemented with 0.003% 1-phenyl-2 thiourea (PTU), until the desired developmental stage. For WISH experiments, embryos were fixed at least overnight in 4% paraformaldehyde (PFA) pH 7.2 in 1 × PBS at 4 °C. For immunofluorescence experiments, embryos were fixed 1.5/2 h at room temperature in 4% PFA pH 7.2 in 1X PBS, to avoid background fluorescence. Following fixation, they were dehydrated with increasing concentrations (30%, 50%, 70%, 100% two times) of methanol (MetOH) in PBT (1X PBS, 0.1% Tween20®) for 5 min. Dehydrated embryos were stored in absolute methanol at -20 °C for at least 1 h or until required. For immunofluorescence experiments requiring phalloidin staining, dehydration in methanol was avoided and embryos were stored in PBT at 4 °C, following PFA fixation.

### Whole mount in-situ hybridization (WISH)

Dig-tagged *smpx* antisense riboprobe was synthesized with the T7 RNA polymerase (Roche) as previously described^17^ from a pre-existent template^31^. *In-situ* hybridization was carried out by using standard procedures on embryos rehydrated with decreasing concentrations (75%, 50%, 25%) of MetOH/PBT for 5 min each, then washed twice with PBT. Minor adjustments to the incubation time in 10 µg proteinase K (pK), for the permeabilization step, were made as follows to avoid potential damages to the neuromasts and PLLp: no pK for 24 hpf embryos, 2.5 min for 29 and 30 hpf embryos, 20 min for 48 hpf embryos, 30 min for 72 hpf larvae and 40 min for 110/120 hpf larvae. PBT washes during the first day were performed with a Tween20® concentration of 0.02% to avoid excessive permeabilization.

### Whole mount immunofluorescence

Dehydrated embryos stored in absolute MetOH at -20 °C were rehydrated with decreasing concentrations (75%, 50%, 25%) of MetOH/PBT for 5 min each, then washed twice with PBT. Embryos were incubated with 10 µg/ml pK in PBS-TT (1X PBS, 0.1% Tween20®, 0.1% Triton X-100 (Sigma-Aldrich)) for a variable time, according to their developmental stage, as described in the previous section. Then, the embryos were rinsed twice for 5 min in 1X PBS, post-fixed with 4% PFA in 1X PBS for 20 min at RT, washed three times with PBS-TT and incubated for 2 h at room temperature in a blocking Solution consisting of 5% bovine serum albumin (Sigma-Aldrich) in PBS-TD (1% DMSO in PBT). Embryos were incubated overnight at 4 °C with primary antibody in Blocking Solution. Primary antibodies and their dilutions used in this work were as follows: rabbit polyclonal anti-SMPX IgG antibody (PA3-070) (ThermoFisher Scientific) 1:100, mouse anti-acetylated α-tubulin (T7451, Merck) 1:200, rabbit anti-GFP antibody (ab290, Abcam). The next day, several washes with PBS-TD were performed, then embryos were incubated overnight at 4 °C with secondary antibody in PBS-TD. The following secondary antibodies and dilutions were used: Alexa Fluor 488 F(ab’)2-Goat anti-rabbit IgG (H + L) Secondary Antibody (A-11070) (ThermoFisher Scientific) 1:200, Alexa Fluor 555 F(ab’)2-Goat anti-mouse IgG (H + L) Secondary Antibody (ThermoFisher Scientific) 1:200, Alexa Fluor 555 F(ab’)2-Goat anti-rabbit IgG (H + L) Secondary Antibody (ThermoFisher Scientific) 1:200. Embryos were counterstained, when required, with 4’,6-diamidino-2-phenylindole (DAPI), 1:2000, to visualize cell nuclei. The last day, several washes in 1X PBS were performed.

### FM 4–64 dye staining of neuromast hair cells

Neuromast hair cells were labelled as described by Erickson and colleagues^29^. Briefly, larvae were incubated in 3 µM FM 4–64 (ThermoFisher Scientific), diluted in embryo water for 60 s, then rinsed at least four time in embryo water.

### Preparation of crRNA:tracrRNA duplex and Cas9 enzyme

Target sites in the zebrafish *smpx* genomic *locus* were found using the ChopChop online tool (https://chopchop.cbu.uib.no), by selecting the crRNAs with the best predicted efficiency and the lowest off-target activity (Supp. Fig. S5A). For the generation of F0 *smpx* knockout embryos, we used the protocol described by Hoshijima and colleagues^42^ with slight modifications. Chemically synthesized *smpx*-specific Alt-R crRNAs (smpx_exon2: 5’-AGGGCTTTGACGTTGGACGA-3’, smpx_prom: 5’-AATAGTCGTGTGATACACAG-3’, smpx_3’UTR: 5’-CAATTCGAATCTCTGAACCA-3’) and universal Alt-R tracrRNA were purchased from Integrated DNA Technologies (IDT). Duplex buffer (IDT) was used to resuspend the two RNA oligos into 100 μM stock solutions that were then aliquoted and stored at -20 °C. The 25 μM duplex cRNA:tracrRNA (dgRNA) was generated by mixing equal volumes of the two 100 μM RNA oligos with duplex buffer under the following annealing protocol: 95 °C for 5 min, cool at 0.5 °C/sec to 25 °C, 25 °C for 10 min, cool to 4 °C rapidly. The 25 μM dgRNA was stored at -20 °C. Alt-R® S.p. Cas9 nuclease, v.3 (IDT) stored as small aliquots at -20 °C. dgRNAs:Cas9 ribonucleoprotein complex (RNP) for microinjection was generated by gently mixing equal volumes of the three 25 μM dgRNAs (to obtain 5 μM of each dgRNA) with nuclease-free water. The Cas9 was used at 15 μM concentration to obtain a 1:1 dgRNAs/Cas9 ratio. These 3 *smpx*-specific dgRNAs (smpx_exon2, smpx_prom, and smpx_3’UTR) were coinjected to generate the crispant larvae used in all the experiments reported in the result section of this paper. Indeed, three synthetic gRNAs were reported to achieve more than 90% biallelic gene knockouts in F0^43^ and for the purpose of this work are believed to increase the efficiency in generating RNA-less embryos.

### Microinjections

Microinjections were performed at the 1-cell stage embryos (for CRISPR/Cas9 experiments we typically used 1-cell stage embryos before cell inflation). The following ATG-targeting morpholino (MO) was used to achieve the repression of *smpx* mRNA translation: 5′-AAGGAAAGTGCTGTTCCCTGGTGTC-3′. MO was diluted in Ringer’s solution (116 mM NaCl, 2.9 mM KCl, 1.8 mM CaCl_2_, 5 mM HEPES, pH 7.3) and injected at a final concentration of 0.3 pmol/embryo in a 4 nL droplet as previously described^11^, using rhodamine or FITC dextran (Sigma-Aldrich) as dye tracer. As a control for each experiment involving *smpx* downregulation, a standard control morpholino (ctrlMO) was injected at the same concentration. For the CRISPR/Cas9 experiments, we typically prepared the injection solution as follows: 1 µL of each of the three 25 µM duplex, 1.23 µL 61 µM Cas9 enzyme, 0.5 µL 5% rhodamine or FITC dextran dye tracer and 0.27 µL nuclease-free water^42^. To test the efficiency of the CRISPR/Cas9 approach, we singularly injected the RNP targeting exon2, or co-injected the 2 RNPs targeted against the promoter and 3’ region (see section “efficiency of the CRISPR/Cas9 approach”) to delete the entire *smpx locus*, maintaining the 1:1 dgRNA/Cas9 ratio. Before microinjecting, the solutions were incubated at 37 °C for 10 min, then left at room temperature; 1 nL of this solution was injected into the yolk. At 24 hpf, the injected embryos were screened and only those exhibiting either the rhodamine- or the FITC-specific fluorescence were used for the experiments. For the CRISPR/Cas9 experiments, uninjected or Cas9-injected embryos were used interchangeably as controls.

### Genomic DNA extraction for indels identification

Genomic DNA extraction was carried out with the zebrafish hotshot method^44^. Briefly, single 48 hpf embryos (at least 5 crispants and 3 controls) were incubated with 50 mM NaOH at 95 °C for 30 min. After cooling down to 4 °C, 1M Tris HCl (pH 8.2) was added to buffer the solution. PCR was performed in 25 µL by using 1 μL of the previously extracted DNA and the following primers: gen_smpx_T7E1_ff (5’-TAGCGTGTGTGTATCCCAGG-3’) and gen_smpx_RevA (5’-CAAGTGTCGACTGTTGGTTAGG-3’) for the RNP targeting exon 2; smpx_DltProm_FF (5’-TCTCTCTTTTCTGAGACCCCAC-3’) and smpx_Dlt3UTR_rr (5’-TCATTTTAAGGTGAACCATCCC-3’) primers were used for the CRISPR/Cas9 experiment aimed at deleting the whole *smpx locus*.

### Efficiency of the CRISPR/Cas9 approach

To assess the mutagenesis capacity of the RNP complex targeting exon 2, an aliquot of the above-described PCR (gen_smpx_T7E1_ff/gen_smpx_RevA) was sequenced (EUROFINS SRL) using the primer gen_smpx_ffA (5’-TAAATACAGAGGCGCAGACAAA-3’), located upstream of the target site. The sanger sequencing results of control and five injected embryos (AB1 files) were uploaded in the free online CRISPR analysis tool Inference of CRISPR Edits (ICE; https://www.synthego.com/products/bioinformatics/crispr-analysis) as previously described^45^. The results of the smpx_DltProm_FF/ smpx_Dlt3UTR_rr PCRs were loaded onto agarose gel and electrophoresis was performed to assess the presence/absence of deletion fragments. In supplementary Fig. 5 is depicted the efficiency of mutagenesis obtained using either the smpx_exon2 dgRNA alone, generating INDEL errors (Supp. Fig. S5B), or the smpx_prom/smpx_3’UTR dgRNAs pair inducing the deletion of the whole *smpx locus* (Supp. Fig. S5C). The ability of the coinjection of the three dgRNAs in generating RNA-less *smpx*^crispant^ embryos was assessed by means of RT-qPCR (Supp. Fig. S5D).

### RNA extraction, cDNA synthesis and qPCR

A minimum of 15 embryos at 24 hpf were pooled and total RNA extracted using the ReliaPrep™ RNA Tissue Miniprep System (Promega) according to the manufacturer’s instructions. cDNA synthesis was carried out by the M-MLV Reverse Transcriptase kit (GeneSpin Srl) starting from 1 μg of total RNA. qPCR was run with the CFX Connect Real-Time PCR detection machine (Bio-Rad Laboratories, Inc.). For each biological replicate, three technical replicates were performed. Briefly, 1 μL of cDNA was amplified in a 20 μL total volume reaction containing a half volume of SYBR Green Master Mix (GeneSpin Srl) and 0.2 μM of each primer. Primer sequences were as follows: RT_smpx_FF (5’-TTTAACACCAGCAGCAGGAAC-3’), RT_smpx_RR (5’-CATCGGGATATTAAGGTTGGCC-3’), rna18s_FF (5’-AGGAATTCCCAGTAAGCGCA-3’), rna18s_RR (5’-ACCTCACTAAACCATCCAATC-3’). Normalization was carried out by analyzing the expression levels of *RNA18s* in parallel with *smpx*. The comparative ΔΔCt method was used to analyze data.

### Imaging

Embryos subjected to WISH, immunohistochemistry, and live larvae subjected to FM 4–64 labeling for neuromast counting/fluorescence quantification were mounted laterally on a glass slide with 80% glycerol (WISH and immunohistochemistry), and 1% low gelling agarose (live larvae). They were imaged and counted singularly by digital camera DFC310FX (Leica) and a Leica Application Suite V4.7 software on a MZFLIII Fluorescence Stereomicroscope (Leica). For embryos hybridized with the *prox1a* RNA probe, PLLp area was measured either by manually drawing the region of interest (ROI) with ImageJ v1.51 or by using the thresholding method. For FM 4–64 dye fluorescence intensity quantification, ImageJ v1.51 was used as previously described^29^. Confocal images were obtained on a Nikon A1 laser-scanning confocal microscope (Nikon Instruments Inc.) and a NIS-Elements C software. Optical sections (Z-stacks) were merged into one maximum projection with Image J. Final panels of images were set up and, when required, adjusted for both contrast and brightness using Adobe Photoshop CS6 (Adobe Systems Inc.).

### Statistical analyses

All the experiments were performed at least twice. For each CRISPR/Cas9 experiment, at least 10 embryos were genotyped at 24 hpf to assess the goodness of *smpx* deletion. For statistical analysis, data were taken from a single experiment and reported as means + / −  sd. Unless specified, biological replicates are the individual embryos analyzed in each experiment. Graphs were generated with GraphPad Prims.

### Supplementary Information

Supplementary Figures.

## Data Availability

All data generated during this study are included in the manuscript.
