# Supplementary material for: Differentiation and functioning of the lateral line organ in zebrafish require Smpx activity
Source: Sci Rep. 2024 Apr 3;14:7862. doi: 10.1038/s41598-024-58138-z (PMC10991396; doi:10.1038/s41598-024-58138-z)
Supplement: Supplementary file 1 — Supplementary Figures. [file 41598_2024_58138_MOESM1_ESM.docx]

**TITLE**

Differentiation and functioning of the lateral line organ in zebrafish require Smpx activity

**AUTHORS**

Alberto DIANA, Anna GHILARDI, Luca DEL GIACCO^(*)^

^(*)^corresponding author

Department of Biosciences, Università degli Studi di Milano, Via Celoria 26, 20133 Milan, Italy

Luca Del Giacco

Tel: +390250314807

E-mail: luca.delgiacco@unimi.it

**Figure S1. Smpx expression in larval neuromasts during development.** Immunofluorescence with the antibody against Smpx (red). The protein labels all neuromasts of 48, 72, 96, 110, and 120 hpf larvae. (A) Confocal Z-stacks of the trunk neuromast L3 immunostained for Smpx and labeled with DAPI (blue) taken from a 48 hpf larva. (B-D) Stereomicroscope reconstructions of whole-mount larvae; lateral views, anterior to the left (note that the image in B was overexposed to reveal the neuromasts signal, which is very weak at this stage) (E,F) Confocal Z-stacks (brightfield + RFP channels) taken from 2 different whole-mount larvae; lateral views, anterior to the left. (F) Magnification of two single neuromasts (L3 and L4) of the PLL.

L1-L7 indicate the neuromasts of the posterior lateral line; the white and green arrowheads mark the position of the anterior lateral line and the accessory neuromasts, respectively; the black arrows point out the terminal neuromasts of the tail.

Scale bars are 4 µm in A, 175 µm in B-E, and 30 µm in F.

**Figure S2. Smpx protein localizes in the cytoplasm and primary cilia of neuromast hair cells.** Double immunofluorescence performed on whole mount 120 hpf larvae with the antibodies against Smpx and acetylated tubulin. Smpx (green, A and C) co-localizes with acetylated tubulin (red, B and C) in the kinocilium, and is arranged in a ‘beads-on-a-string’ fashion (white arrowheads). (C) Merge of the two signals (orange). Confocal Z-stacks taken from whole-mount larvae; lateral views, anterior to the left. Larvae were counterstained with DAPI (blue) to visualize cell nuclei.

Scale bars are 17 µm.

**Figure S3. The larvae of the *tg (sod1:sod1; hsp70:DsRed)* transgenic line express the DsRed fluorescent protein in the outermost cells of neuromasts.** Representative neuromast of the double transgenic line *Tg (sod1:sod1; hsp70:DsRed)* ; *(cldnb:GFP)*; confocal Z-stacks of whole-mount 120 hpf larva. (A) The GFP signal labels all the cell types of the neuromast. (B) The DsRed signal is restricted to a peripheral cell population. (C) Result of merge of GFP and DsRed signals showing that the cells expressing the DsRed are those confined to the outermost region of the mature neuromast (orange).

Scale bar is 50 µm.

FM 4-64

FM 4-64

**Figure S4. Smpx-deficiency affects the number of intact neuromasts.** (A,B) Representative stereomicroscope images of the PLL organ of whole-mount live larvae incubated with the FM 4-64 vital dye. (A) A control larva displaying the proper number of intact neuromasts (white arrowheads). (B) The number of intact neuromasts (white arrowheads), identified through their mature/functional HCs, is dramatically lowered by the lack of Smpx. (C) Quantification of the number of intact neuromasts in *smpx*^crispant^ (n = 22) vs ctrl larvae (n = 18). Confocal Z-stacks of (D) control and (E) *smpx*-crispant larvae of the *Tg (sod1:sod1; hsp70:DsRed)* line. (F) Quantification of the number of neuromasts in *smpx*^crispant^ (n = 37) vs ctrl larvae (n = 26).

Lateral views, anterior to the left. Scale bars are 120 µm in A and 500 µm in D.

**Figure S5. Efficiency of the CRISPR/Cas9 approach.** (A) Genomic organization of zebrafish *smpx*. For the RNP targeting exon 2, the primers (blue arrows) flanking the target site (black scissor) and the primer used for sequencing (red arrow) are indicated. For the RNPs targeting the promoter and the 3’UTR of zebrafish *smpx,* the primers (black arrows) flanking the two target sites (red and blue scissors) are indicated. (B) Quantification of INDEL percentage with the RNP targeting exon 2. Five 24 hpf injected embryos were randomly selected and the target region of each one of them was amplified, sequenced, and analyzed for indel percentage with the ICE online tool (Synthego). (C) Representative agarose gel band resulting from a PCR with genomic DNA extracted from a single 24 hpf embryo, and primers flanking the promoter region and the 3’UTR of zebrafish *smpx*, respectively. The entire *smpx* chromosomal locus was effectively deleted as demonstrated by the presence of the junction fragment whose size of ~270 bp was consistent with the deletion of the whole gene. Five individual injected larvae were analyzed and resulted positive for the presence of deletion fragments (original agarose gel image available upon request). (D) RT-qPCR showing that the amount of *smpx* mRNA dramatically decreases following CRISPR/Cas9 gene knockout. Primers for qPCR were designed to flank the cut site within exon 2. Data are representative of three independent experiments.
